# Higher order *Arabidopsis* 14-3-3 mutants show 14-3-3 involvement in primary root growth both under control and abiotic stress conditions

**DOI:** 10.1093/jxb/eru338

**Published:** 2014-09-04

**Authors:** P. J. M. van Kleeff, N. Jaspert, K. W. Li, S. Rauch, C. Oecking, A. H. de Boer

**Affiliations:** ^1^Faculty of Earth and Life Sciences, Department of Structural Biology, Vrije Universiteit, De Boelelaan 1085, 1081 HV Amsterdam, The Netherlands; ^2^Centre for Plant Molecular Biology—Plant Physiology, University of Tübingen, Auf der Morgenstelle 32, 72076 Tübingen, Germany; ^3^Faculty of Earth and Life Sciences, Department of Molecular and Cellular Neurobiology, Centre for Neurogenomics and Cognitive Research (CNCR), Vrije Universiteit, De Boelelaan 1085, 1081 HV Amsterdam, The Netherlands

**Keywords:** Redundancy, 14-3-3, abiotic stress, LEH, stress-interactome, primary root.

## Abstract

*Arabidopsis* 14-3-3 proteins are a family of conserved proteins that interact with numerous partner proteins in a phospho-specific manner, and can affect the target proteins in a number of ways; e.g. modification of enzymatic activity. We isolated T-DNA insertion lines in six 14-3-3 genes within the non-epsilon group that phylogenetically group in three closely related gene pairs. In total, 6 single, 3 double, 12 triple, and 3 quadruple mutants were generated. The mutants were phenotyped for primary root growth on control plates: single and double mutants were indistinguishable from WT, whereas six triples and all quadruples showed a shorter primary root. In addition, length of the first epidermal cell with a visible root hair bulge (LEH) was used to determine primary root elongation on medium containing mannitol and 1-aminocyclopropane-1-carboxylic acid (ACC). This analysis showed clear differences depending on the stress and 14-3-3 gene combinations. Next to the phenotypic growth analyses, a 14-3-3 pull-down assay on roots treated with and without mannitol showed that mannitol stress strongly affects the 14-3-3 interactome. In conclusion, we show gene specificity and functional redundancy among 14-3-3 proteins in primary root elongation under control and under abiotic stress conditions and changes in the 14-3-3 interactome during the onset of stress adaptation.

## Summary

Our research shows that there is isoform specificity and redundancy among 6 out of 13 14-3-3 members in root growth under control and abiotic stress conditions.

## Introduction

**Fig. 1.**
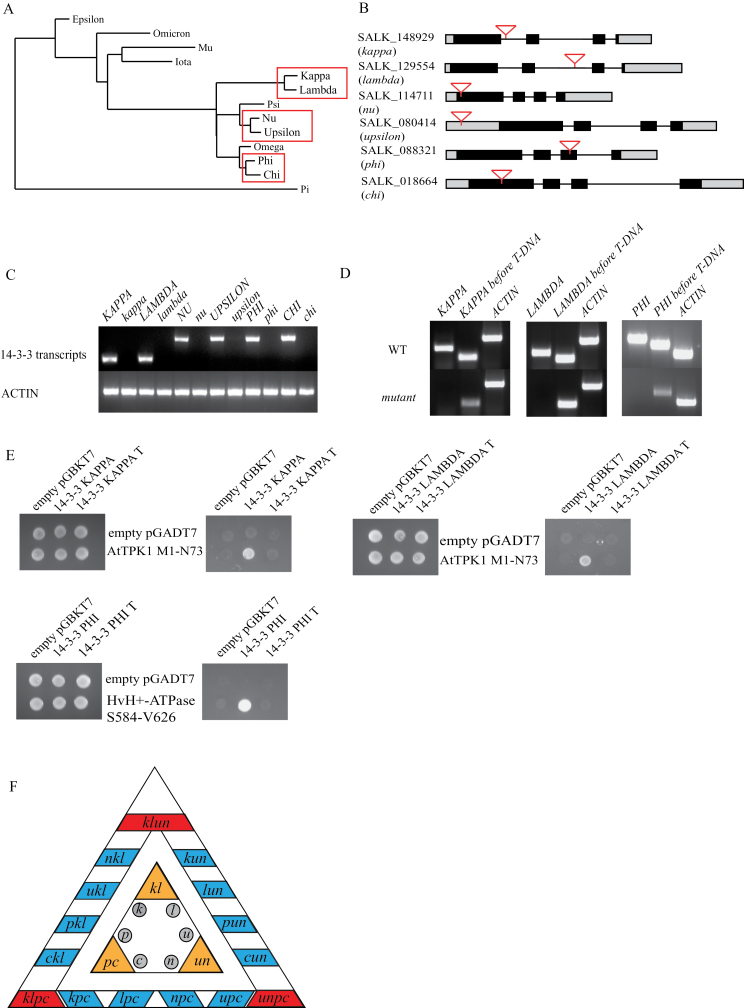
14-3-3 T-DNA insertion mutants. (A) Phylogenetic tree of *Arabidopsis* 14-3-3s with the three closely related gene pairs of which T-DNA insertion lines are used in this study shown in boxes. (B) Position of T-DNA insertion within the six *Arabidopsis* 14-3-3 genes used in this study. Grey boxes represent UTRs, black boxes exons, lines represent introns, and inverted triangles indicate T-DNA insertion. (C) Expression of 14-3-3 in insertion mutants. Total RNA was isolated from 14 day after stratification (DAS) plants and RT-PCR was used to amplify 14-3-3 transcripts. No full length 14-3-3 transcripts are found in the single mutants. (D) In-frame truncated transcripts have been found in the single mutants *kappa*, *lambda*, and *phi*. (E) Yeast-2-hybrid (Y2H) assay using wild-type 14-3-3s and truncated 14-3-3s (14-3-3 T) showing that the truncated 14-3-3s do not bind to 14-3-3 target proteins, whereas wild-type 14-3-3 does. (F) Overview of the mutants used and generated during this study. The inner triangle depicts the single mutants in grey and at the corners in orange are the double mutants. The outer triangle shows at the sides in blue 12 triple mutants and at the corners in red the quadruple mutants.

14-3-3 proteins act within protein phosphorylation cascades by associating to specific phospho-sites. The outcome of 14-3-3 binding to their targets depends upon the target itself: i.e. it can change enzyme activity, prevent or induce protein degradation, or alter the subcellular localization of proteins (Igarashi *et al.* 2001; MacKintosh *et al.* 2001; Sirichandra *et al.* 2010). 14-3-3 proteins bind to phosphorylated targets as homo-/heterodimers through three canonical binding motives: internal mode-I ((R/KXXpS/pTXP), internal mode-II (R/KXXXpS/pTXP), and C-terminal mode-III pS/pTX_1-2_-COOH) (de Boer *et al.* 2013). *Arabidopsis* expresses 13 14-3-3 members, which can be grouped based on their amino acid sequence into an epsilon and non-epsilon group (Fig. 1A) (Jaspert *et al.* 2011; Paul *et al.* 2012; de Boer *et al.* 2013). The epsilon group is found in all eukaryotic organisms and is most likely necessary for basal eukaryotic functions. The non-epsilon group members potentially have organism-specific functions (Jaspert *et al.* 2011).

The 14-3-3 proteins and their targets have been shown to play a role in primary metabolism, stress related pathways, and hormone-controlled plant development (Cotelle *et al.* 2000; Yan *et al.* 2004; Sirichandra *et al.* 2010; Jaspert *et al.* 2011; de Boer *et al.* 2013; Yoon *et al.* 2013). Analysis of the 14-3-3 interactome in *Arabidopsis* showed the presence of many target proteins functioning in various primary metabolic pathways, including nitrate, carbon, and sulphur metabolism (Chang *et al.* 2009; Diaz *et al.* 2011; Shin *et al.* 2011). Well-known 14-3-3 interactors involved in metabolism are nitrate reductase (NR) (MacKintosh *et al.* 2001), the plasma membrane localized H^+^-ATPase (Svennelid *et al.* 1999), and sucrose-phosphate synthase (SPS) (Toroser *et al.* 1998).

Abiotic stress induces stress-related protein phosphorylation and 14-3-3 proteins have been shown to bind to these distinct stress-related targets (Schoonheim *et al.* 2009; Sirichandra *et al.* 2010). One group of well-known interactors with a function in osmotic stress adaptation is the AREB/ABF bZIP transcription factor family. In *Arabidopsis*, ABF3 becomes phosphorylated at T451 by SnRK2.6, creating a 14-3-3 binding site, where subsequent 14-3-3 interaction prevents ABF3 protein degradation (Sirichandra *et al.* 2010).

To date, few phenotypes have been described for *Arabidopsis* 14-3-3 mutants and the phenotypes described demonstrate the involvement of 14-3-3 proteins in plant development. The ectopic overexpression of wheat 14-3-3 genes (Ta14-3-3) in *Arabidopsis* resulted in shorter primary roots, delayed flowering, and retarded growth rates (Li *et al.* 2013). The single mutants of At14-3-3 *mu* and *upsilon* both show a delayed flowering phenotype under long-day conditions (Mayfield *et al.* 2007). However, only *mu*, and not *upsilon*, shows a reduction in root growth, compared to WT, under continuous light (Mayfield *et al.* 2012). Another example of 14-3-3 isoform specificity was found between the closely related proteins KAPPA and LAMBDA. Only LAMBDA is necessary for PHOT2-mediated stomatal opening, whereas KAPPA did not show an effect (Tseng *et al.* 2012).

Similarly to other multi-gene families, the 14-3-3 family has been the subject of debate over whether or not there is redundancy. 14-3-3 redundancy occurs in S*accharomyces cerevisiae*, which has two 14-3-3 isoforms. Mutating both isoforms is lethal (van Heusden *et al.* 1995). This lethal phenotype is rescued by adding one 14-3-3 gene of another species, such as plant or human (van Heusden *et al.* 1996). This shows, in addition to redundancy, that 14-3-3 proteins of different species can functionally replace each other. To answer the question of redundancy in *Arabidopsis*, we isolated T-DNA insertion mutants for six of the eight members of the non-epsilon group. Phylogenetic analysis grouped them as three closely related gene pairs. A total of 24 mutants were generated. Overall, we show isoform specificity and redundancy (full and unequal) in primary root growth and root cell elongation under various abiotic stresses. In addition, an *in vivo* pull-down revealed that the 14-3-3 interactome changes greatly upon osmotic stress treatment (mannitol) of *Arabidopsis* roots.

## Material and methods

### Plant growth and material

*Arabidopsis thaliana* Columbia ecotype (Col-0) seeds were surface sterilized by rinsing the seeds in 70% ethanol (10min), followed by 10min 25% bleach + 0.1% Tween-20. Thereafter, the seeds were washed three times with sterilized MQ and resuspended in 0.1% sterile agarose. Seeds were plated on 0.5×MS medium (pH 5.8) solidified with 12g l^–1^ of plant agar (Sigma A1296) and stratified for 3 d at 4 °C. For germination, plates were placed vertically in a growth chamber with 14h light (22 °C)/10h dark (18 °C), 170 µmol m^–2^ s^–1^. Salk-line insertion mutants were isolated for *KAPPA* (AT5g65430; SALK_148929), *LAMBDA* (AT5g10450; SALK_129554), *NU* (AT3g02520; SALK_114711), *UPSILON* (AT5g16050; SALK_080414), *PHI* (AT1g35160; SALK_088321), and *CHI* (AT4g09000; SALK_018664). From the single mutants, the double mutants *kl*, *un*, and *pc* were generated (according to their closely related gene pair). Triple and quadruple mutants were generated by crossing the double mutants. T-DNA insertions were verified by PCR (primers in Supplementary Table S1a, available at JXB online).

### Root RNA isolation and RT-PCR

Seeds were sterilized as mentioned above. Four days after stratification (DAS), WT seedlings were transferred to 120mm × 120mm petri dishes containing 0.5×MS medium (pH 5.8) solidified with 12g l^–1^ of plant agar (Sigma A1296) (=day 4). At day 7 and 14, roots were harvested. RNA was isolated using Total RNA isolation form plant (NucleoSpin® RNA plant) according to the manufacturer’s manual. 1 µg of RNA, an oligo(dT) primer, and SuperScript™-II Reverse Transcriptase (Invitrogen) was used to convert RNA into first strand cDNA. Primers and PCR conditions for 14-3-3 transcripts can be found in Supplementary Table S1b (available at JXB online).

### Root growth assay

Seeds were sterilized as mentioned above. Four d after stratification (DAS), 2 seedlings of WT and 2 seedlings of mutant plants were transferred to 120mm × 120mm petri dishes containing 0.5×MS medium (pH 5.8) solidified with 12g l^–1^ of plant agar (Sigma A1296) with or without abiotic stress (=day 4). Plates were scanned using a flatbed scanner at day 7, 9, 11, and 14 after transfer. Root phenotypes were analysed using EZ-Rhizo (Armengaud *et al*. 2009). Statistical analysis was performed in SPSS (version 21).

### Root cell length measurement using the length of the first epidermal cell with a visible root hair bulge (LEH)

The LEH was first described in Lee *et al*. 2001 and is the value of epidermal cell length of trichoblast cells, which is very stable. Seeds were surface sterilized as described previously. Four DAS seedlings were transferred to 0.5×MS-0.5% sucrose solidified with 12g l^–1^ of plant agar (Sigma A1296) and further grown vertically under the same conditions for 3 d. Typically 12 plants per genotype were transferred to 0.5×MS-0.5% sucrose in the presence or absence of abiotic stress (200mM mannitol and 5 µM 1-aminocyclopropane-1-carboxylic acid (ACC)). After transfer the plates are placed back, vertically, into the growth chamber. Pictures were made 3h after treatment with a stereomicroscope (Leica MZPLIII). Distances between the first visible root hair bulge from the tip and the root hair of the adjacent cell in the same cell file were measured using ImageJ (Schneider *et al.* 2012). The LEH value is the average value of three independent experiments and is set relative to WT. For easier interpretation of the data, the mutant LEH value is subtracted from the WT value. Statistical analysis was performed in SPSS (version 21).

### Atomic absorption spectroscopy (AAS)

Plants were harvested from plate and dried overnight at 80 °C. Per 1mg of dry material 400 µl of MQ water was added and samples were boiled for 1h. Supernatant was filtered using Acrodisc® 0.2 µm PITE syringe filters. Na^+^ and K^+^ concentrations were determined by atomic absorption spectroscopy (Perkin Elmer AAS100).

### Root extract 14-3-3 pull-down assay

Seeds were sterilized, stratified, and germinated on plates as mentioned above. Eleven d after stratification (DAS) plants were transferred to 0.5×Hoagland (pH 5.5) and grown in a growth chamber with 14h light (22 °C)/10h dark (18 °C), 170 µmol m^–2^ s^–1^. At day 31 the plants were treated with 200mM mannitol for 0h, 10min, and 3h. The roots were harvested, dried with paper tissue, and weighed, after which they were snap frozen in liquid nitrogen.

Total root protein was extracted by grinding the plant material in liquid nitrogen after which per gram root material, 2ml ice-cold extraction buffer was added (50mM HEPES-NaOH (pH 7), 10mM MgCl_2_.6H_2_O, 1mM Na_2_-EDTA, 2mM DTT, 10% ethylene glycol, 0.02% Triton, 1 tablet protease inhibitor EDTA free (Roche) per 30ml buffer and 1 tablet PhosSTOP (Roche) per 10ml buffer). The extract was centrifuged twice for 20min at 20 000 *g* at 4 °C. Protein concentration was measured using Bradford assay (Biorad) and a total of 5mg protein per 2ml root extract was used per pull-down assay.

PureProteome™ Nickel magnetic Beads (Millipore) were coated with 50 µg of total recombinant His-14-3-3 (10 µg His-KAPPA, 10 µg His-LAMBDA, 10 µg His-NU, 10 µg His-UPSILON, and 10 µg His-PHI) according to the manufacturer’s protocol. Per pull-down assay 100 µl of coated beads were incubated with the root extract while gently rocking overnight at 4 °C. The beads were washed in 1ml wash buffer containing 10mM imidazole for 5min five times. Thereafter, the beads were eluted for 20min with 100 µl 100 µM NIP (a peptide that cannot interact with 14-3-3; ERYMGICMRKQYNNFVPVCLRS) minus 10mM imidazole (to reduce the background protein loss), followed by 100 µl 100 µM R18 peptide (PHCVPRDLSWLDLEANMCLPP) in wash buffer without imidazole (De Vries-van Leeuwen *et al.* 2013). R18 is a peptide with high affinity for the 14-3-3 groove. Thereafter, the 14-3-3 beads were eluted with 100 µl wash buffer containing 50mM imidazole for 20min. 10% SDS-page gel was loaded with 25 µl of NIP, R18, and imidazole elutions. Gels were stained with Coomassie (Bio-Rad) and each gel lane was cut in four slices. The gel slices were transferred to 1.5ml eppendorf tubes and cut in smaller pieces. All incubation steps were performed while vortexing unless stated otherwise. The gel particles were incubated for 20min in 1ml 25mM NH_4_HCO_3_/50% acetonitrile to destain the gel particles. This step was repeated until the gel particles were transparent. The solution was removed and gel particles were incubated for 20min in 100 µl 100% acetonitrile. Supernatant was removed and gel particles were dried in speed-vac for maximum 30min. 1ml of 10mM dithiothreitol (DTT) was added and incubated for 1h at 56 °C without vortexing, cooled to RT, and replaced by 1ml of 55mM iodoacetamide (IAM) and incubated for 45min in the dark with vortexing every 15min. The gel particles were washed twice for 10min with 1ml 50mM NH_4_HCO_3_ followed by one wash for 10min with 25mM NH_4_HCO_3_/50% acetonitrile. Thereafter, the gel particles were dried in the speed-vac and stored at –80°C. Gel particles were thawed and re-swelled with 10ng ml^–1^ sequencing grade modified trypsin (Promega) in 50mM NH_4_HCO_3_ for 45min at 4 °C enough to cover gel particles. Afterwards, 20 µl of 50mM NH_4_HCO_3_ was added to cover the pieces and digested overnight at 37 °C. Peptides were extracted twice in 0.1% acetic acid/50% acetonitrile for 20min while vortexing; the peptides were dried in Speed-vac and dissolved in 50 µl 0.1% acetic acid in HPLC water and vortexed for 20min. Samples were cleared from gel particles using a 0.45 µm low-protein-binding spin column (Millex-HV) and samples were run on MALDI-TOF-MS (ABI5800). MS/MS spectra were searched against an IPI *Arabidopsis* database (ipi.ARATH.v3.85) with the ProteinPilotTM software (version 3.0; Applied Biosystems, Foster City, CA, USA; MDS Sciex) using the Paragon algorithm (version 3.0.0.0) as the search engine. The search parameters were set to cysteine alkyation with acrylamide, and digestion with trypsin. Detected protein threshold was set in protein summary to 0.5 achieving a 95% confidence.

## Results

### Isolation of 14-3-3 T-DNA insertion mutants.

To study the function of non-epsilon 14-3-3s, T-DNA insertion lines were isolated for six of the eight non-epsilon 14-3-3 genes; *KAPPA* (*K*), *LAMBDA* (*L*), *NU* (*N*), *UPSILON* (*U*), *PHI* (*P*), and *CHI* (*C*) (excluded are OMEGA and PSI). These six genes were chosen based on a phylogenetic tree that shows that these genes form three groups of closely related gene pairs, *K*-*L*, *U*-*N*, and *P*-*C* (Fig. 1A). The presence and location of the T-DNA within the 14-3-3 genes were confirmed by PCR and sequencing (Fig. 1B). The presence of a T-DNA insertion within a gene does not guarantee that these lines are null-mutants. By means of semi-quantitative reverse transcriptase PCR (RT-PCR), we confirmed that full length *KAPPA*, *LAMBDA*, *NU*, *UPSILON*, *PHI*, and *CHI* transcripts are not present in the respective T-DNA insertion lines (Fig. 1C). Although *kappa*, *lambda*, and *phi* do not express full-length transcripts, in-frame transcripts were found covering the open reading frame before the T-DNA insertion (Fig. 1D). These truncated transcripts could be translated into truncated but partially functional proteins. For example C-terminal truncation mutants of *Arabidopsis* 14-3-3OMEGA have shown an increased affinity for target proteins (Shen *et al.* 2003). To study the binding properties of these truncated 14-3-3s, a yeast-two-hybrid assay (Y2H) with known interactors was performed. For KAPPA and LAMBDA the N-terminus of the vacuolar ion channel TPK1 was used (Latz *et al.* 2007; Voelker *et al.* 2010), and the barley H^+^-ATPase was chosen as PHI target protein. Figure 1E shows that the possible truncated versions of *KAPPA*, *LAMBDA*, and *PHI* do not bind 14-3-3 target proteins in a Y2H assay. This result indicates that the truncated KAPPA, LAMBDA, and PHI do not bind with target proteins. Therefore, we conclude that the T-DNA insertions in the six 14-3-3 genes result in loss-of-function mutants. Higher order mutants were generated to examine the genetic interactions among the six 14-3-3 genes. Included in the higher mutants are double mutants between each highly similar pair (*kl*, *un*, *pc*), 12 triple mutants and 3 quadruple mutants which were generated using the double mutants as background (Fig. 1F and Supplementary Fig. S1, available at JXB online).

### Root phenotypes of 14-3-3 mutants under control conditions.

**Fig. 2.**
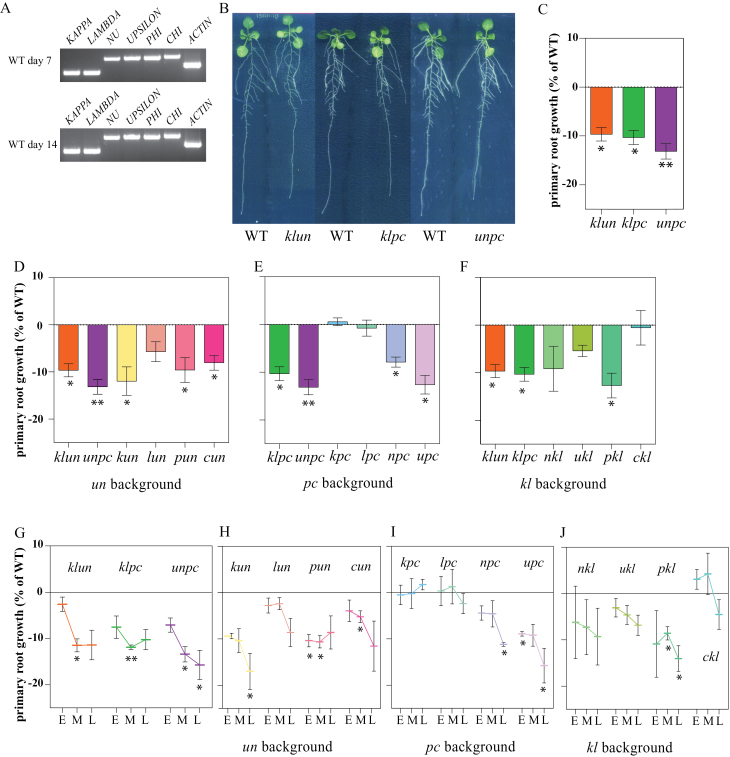
Primary root growth of 14-3-3 triple and quadruple mutants during 7 d shows mutant specific inhibition and time-dependent growth. (A). 14-3-3 *KAPPA*, *LAMBDA*, *NU*, *UPSILON*, *PHI*, and *CHI* expression in roots at 7 and 14 d after stratification (DAS) determined by RT-PCR. (B–J) Two wild-type (WT) and two mutant seedlings were grown vertically on plates under long-day conditions and root growth was measured as described in the materials and methods. Figures show mutant root growth data compared with the WT on the same plate. (B) 14-3-3 quadruple mutants exhibited a shorter primary root compared with WT at 14 DAS. (C) Primary root growth between 7 and 14 DAS of the three 14-3-3 quadruple mutants depicting shorter mutant roots. (D) Three triple mutants in the *un* background (*kun*, *cun* and *pun*) show a reduction in primary root growth. (E) Primary root growth for *pc* background plants shows that the triples *npc* and *upc* are impaired, whereas *kpc* and *lpc* are indistinguishable from WT. (F) Primary root growth for triple mutants in the *kl* mutant background shows that only the growth of *pkl* is significantly reduced. (G–J) Primary root growth rates were calculated between two measuring days: DAS 7–9 or early (E), 9–11 or middle (M), and 11–14 or late (L) show overall a time-dependency in growth for most triple and quadruple mutants. (G) Quadruple mutants *klun*, *klpc*, and *unpc* show an inhibition in growth rate. (H) *kl* background; *pkl* is inhibited in PR length in both middle and late growth period, (I) *un* background plants showing *pun* and *cun* to be inhibited between day 9 and 11, (J) *pc* background plants where *upc* and *npc* show a growth inhibition between day 11 and 14. (K) Overview of the primary root growth of the 24 14-3-3 mutants on control plates. Blue coloured boxes show a significant difference compared with WT. Bars show average of three biological replica’s and the error bars depicts SEM. Statistical analysis paired t-test *<0.05 *P*>0.01, **<0.01 *P*>0.005,*** *P*<0.005.

14-3-3 proteins have been linked to differences in primary root length in various plant species (Mayfield *et al.* 2012; Li *et al.* 2013; Yang *et al.* 2013). Primary root (PR) growth was measured between 7 and 14 d after stratification (DAS). To validate the expression of the six 14-3-3s during the root growth assay, their transcripts were detected by RT-PCR (Fig. 2A). This analysis shows that all six 14-3-3s are indeed expressed in roots during the course of the experiment. The single and double mutants were indistinguishable from WT in relation to the primary root length at any of the time points (data not shown). However, the PR length at 14 DAS of the three quadruple mutants was significantly reduced as compared with WT (8–11%; Fig. 2B, C). To dissect which combinations of 14-3-3s could cause a similar effect, all 12 triple mutants were set on a plate. The results were grouped according to their double mutant background to enable the comparison of different added mutations. To ensure that the reduced PR length seen was not due to root length differences at the start of the assay, PR length was calculated between day 7 and 14 (Fig. 2D–F, K). The background to which mutations are added determine the growth phenotype. The growth reduction of the *klun* quadruple mutant is solely due to the *kun* combination, because in the *un* background the *kappa* mutation alone results in a significant growth reduction (Fig. 2D; *kun*), whereas *nkl*, *ukl*, and *lun* do not. The addition of *phi* and *chi* in the *un* background and *nu* and *upsilon* in the *pc* background shows a dosage-dependent effect between these four genes as reduced root growth is seen in all triple mutants and in the quadruple mutant *unpc* (Fig. 2D, *pun*, *cun*; E, *npc*, *upc*). The *pc* background (Fig. 2E) shows no reduced primary root growth for *kappa* and *lambda* in the *pc* background. In the *kl* background, *phi* does contribute to the growth reduction of the *klpc* quadruple mutant, whereas the closely related *chi* does not (Fig. 2F). Consequently, the reduced root growth phenotype of *klpc* can be traced back to the *klp* combination. The mutant background is very important: e.g. 14-3-3*phi* has an isoform specific role with respect to 14-3-3*chi* in primary root growth in the *kl* background, but in the *un* background the contribution of both 14-3-3*phi* and 14-3-3*chi* are equal showing a dosage dependence (Fig. 2D, F, K).

### Time-dependency in root growth

As root growth inhibition was seen between 7 and 14 d in the higher order mutants, we further dissected root growth rates in this period to establish a time point of deviation between the mutants and WT. Therefore, we evaluated the growth rates during two measuring days: day 7–9 (early), 9–11 (middle), and 11–14 (late) (Fig. 2G–J). As shown in these figures, the effect of the mutants on root growth is time-dependent. Among the quadruple mutants the growth inhibition of *unpc* steadily increases over the seven day measuring period (Fig. 2G), whereas the inhibition in the *klpc* and *klun* mutant is strongest in the middle section (day 9–11). Similarly to the quadruple mutants, most triple mutants show time dependency in root growth with the strongest decrease in growth rates in the late part of root growth (Fig. 2H–J). So, for the PR growth of 14-3-3 mutants we observe time-dependency, isoform specificity, and isoform-specific redundancy.

### Double mutant *un* shows reduced PR growth inhibition on salt and mannitol

**Fig. 3.**
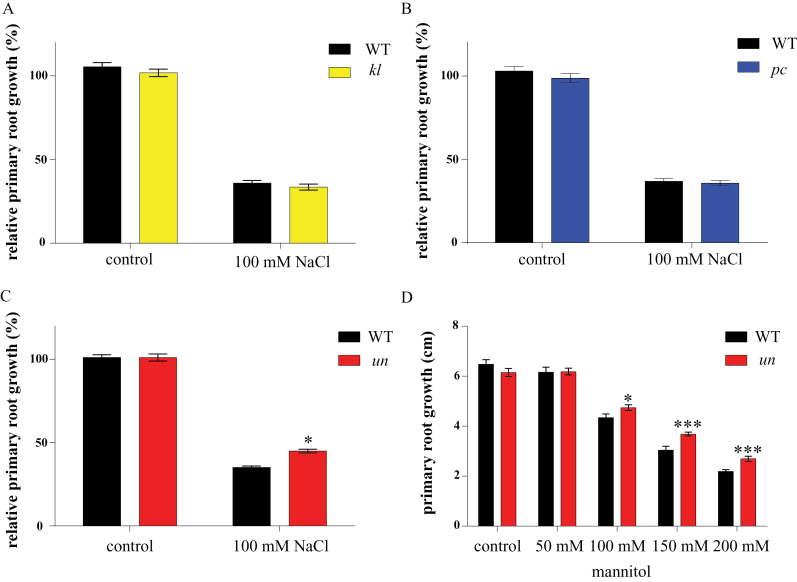
Double 14-3-3 mutant primary root phenotypes on 100mM NaCl and different mannitol concentrations. Plants were grown on 0.5× MS plates for 4 DAS after which they were transferred to medium with or without 100mM NaCl or different concentrations of mannitol and grown an additional 10 d. Root growth was measured using EZ-Rhizo. Main root growth is total length day 10 – total length day 3 set relative to control root growth. (A, B) Root growth of *kl* and *pc P*≥0.05. (C) Root growth ofr *un*. *P*<0.05 for 2-way ANOVA interaction. Bars indicate SEM; *n*=3. (D) Main root growth of double mutant *un* on different concentrations of mannitol. Bars indicate SEM 2-way ANOVA interaction *P*=0.005 and *P*=0.003 for 150mM and 200mM, respectively (*n*=2).

To elucidate the possibility of 14-3-3 involvement in root growth during abiotic stress, a pilot plate assay was performed. For the pilot assay, WT, *kl*, *un*, and *pc* seedlings were grown for 10 d on plates with and without 100mM NaCl. As described above, on control plates no difference was seen between the double mutants and WT. However, the double mutant *un* showed less reduction in primary root growth on 100mM NaCl compared with WT (2-way ANOVA interaction *P*<0.05), whereas the other two double mutants (*kl* and *pc*) were indistinguishable from WT (Fig. 3A–C). To determine if this reduced sensitivity to salt is due to differences in ion homeostasis, atomic absorption spectroscopy (AAS) was used to analyse the sodium (Na^+^) and potassium (K^+^) content of 14 DAS double mutant plants grown for 10 d on 0.5×MS agar plates containing 100mM NaCl. No significant differences in ion content were found either in the control situation or plants grown on 100mM NaCl (data not shown) (2-way ANOVA interaction *P*>0.05 for all). This raises the question whether the reduced salt sensitivity phenotype of the *un* double mutant is caused by the osmotic component of salt stress. To address this question a plate assay supplemented with different concentrations of mannitol (0–200mM) was performed. Figure 3D shows that on mannitol plates supplemented with 150mM and 200mM mannitol (100mM NaCl is iso-osmotic with approx. 200mM mannitol (Torabi and Niknam, 2011), *un* grows similar as on NaCl plates (Fig. 3D). This data, combined with the AAS data, illustrates that the reduced sensitivity of primary root growth of *un* found on 100mM NaCl is due to osmotic stress and not ionic stress, hence linking the 14-3-3s to the osmotic stress pathway.

### Isoform specificity among the 14-3-3s to different abiotic stresses

**Fig. 4.**
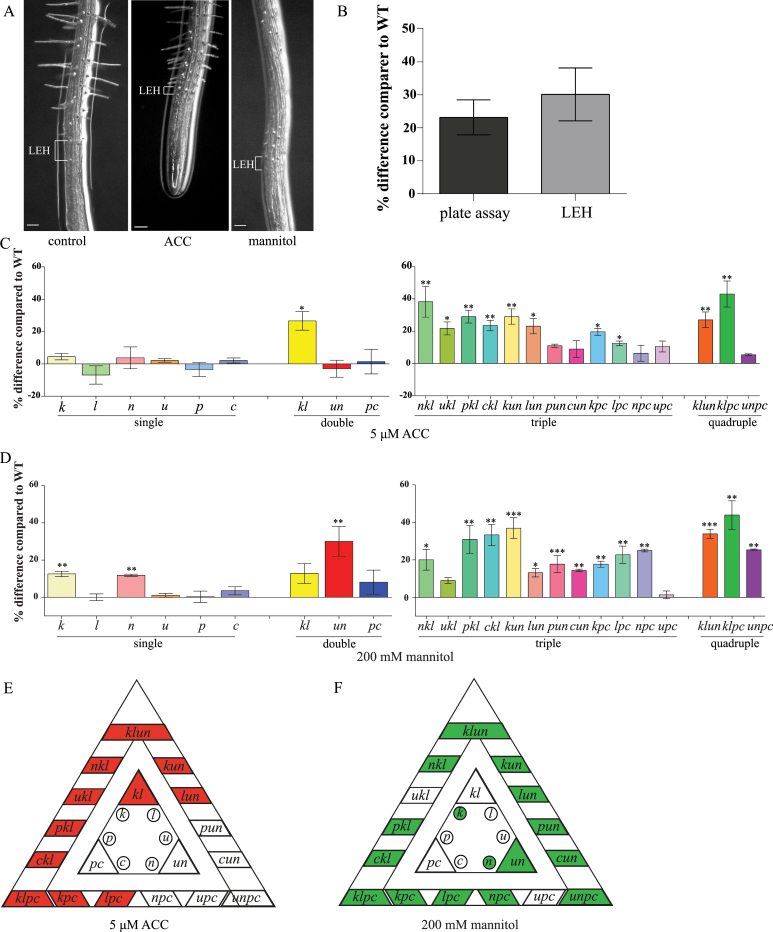
Root epidermal cell elongation (LEH) of 14-3-3 mutants on 5 µM ACC and 200mM mannitol after 3h. WT was set to 100% and the mutants are relative to WT. Percentage difference was calculated as mutant – WT. (A) Representative pictures of WT LEH on control, 200mM mannitol, and 5 µM ACC. Scale bar is 100 µm. (B) Comparison between LEH and plate assay for the double mutant *un* showing similar percentage differences compared with WT. (C) LEH of single, double, triple, and quadruple mutants on 5 µM ACC. (D) LEH of single, double, triple, and quadruple mutants on 200mM mannitol. (E, F) Overview of LEH results of the 24 14-3-3 mutants on 5 µM ACC (red) and 200mM mannitol (green). Coloured boxes show a significant difference compared with WT. The significance level can be seen in figure C–E. * *P*<0.05, ** *P*<0.01, *** *P*<0.005; error bars indicate SEM (*n*=3).

The plate assay data indicates isoform specificity or redundancy among the 14-3-3s. To test this possibility the 6 single, 3 double, 12 triple, and 3 quadruple 14-3-3 mutants were screened using LEH (Le *et al.* 2001; Cnodder *et al.* 2007). LEH (the length of the first epidermal cell with a visible root hair bulge) is a quantitative method to measure fast changes in cell elongation using the size of the trichoblasts (trichoblast is a hair-forming cell on the epidermis of the plant root) at the moment of root hair formation (Le *et al.* 2001; Cnodder *et al.* 2007; Staal *et al.* 2011; Tsang *et al.* 2011). Even though LEH is measured after 3h, it correlates to primary root elongation under long-term stress for ACC (Le *et al.* 2001). As this assay was first developed for ACC, we measured the LEH on ACC for the 14-3-3 mutants. In addition, the phytohormone ethylene is elevated upon osmotic stress perception (Felix *et al.* 2000; Zhao *et al.* 2004). A precursor of ethylene, 1-aminocyclopropane-1-carboxylic acid (ACC), induces a reduction in LEH in WT *Arabidopsis* roots as compared with plants on control plates (Fig. 4A) (Le *et al.* 2001; Cnodder *et al.* 2007; Staal *et al.* 2011; Tsang *et al.* 2011). The *kl* double mutant showed a 27% increase in LEH on ACC plates compared with WT (Fig. 4C). At the single mutant level, neither *kappa* nor *lambda* differed significantly from the WT, indicating redundancy (Fig. 4C). The quadruple mutants in the *kl* background (*klun* and *klpc*) showed a larger LEH compared with WT but similar to the *kl* double mutant (2-way ANOVA interaction *P*>0.05), whereas *unpc* was indistinguishable from WT (Fig. 4C). Only the triple mutants in the *kl* background or those mutants that contained either *kappa* or *lambda* (*kun*, *lun*, *kpc*, and *lpc*) showed a similar increase in LEH on ACC as *kl* (Fig. 4C, E). This shows that both *un* and *pc* enhance the *kappa* and *lambda* single mutant phenotype, but that *un* and *pc* themselves are not involved in ACC as *unpc* is indistinguishable from WT. These results indicate that both *kappa* and *lambda* are involved in reduced sensitivity towards exogenous ACC and they show redundancy.

### Antagonistic effects for *upsilon* in the *kl*- and *pc*-background for the LEH on mannitol

The robustness of the LEH compared with primary root growth on plates was tested for *un* on 200mM mannitol. Indeed, both on plate as well as in the LEH assay, root growth of *un* mutants was 23% (on plate) and 30% (in LEH) larger than that of WT roots, whereas growth of the other double mutants was indistinguishable from WT (Fig. 4B, D). On mannitol all quadruple mutants, *klun*, *klpc*, and *unpc*, showed a significant larger LEH compared with WT (30, 44, and 25%, respectively), which indicated that *un* is not the only mutant background in which reduced sensitivity to mannitol occurs (Fig. 4D, F). Next, we measured the LEH on mannitol for the triple mutants. In the *kl* background, addition of the *upsilon* mutation (= *ukl*) had no effect on LEH, whereas addition of each of the other three remaining mutations (*nu*, *phi*, and *chi*) resulted in LEH phenotypes similar to that of *un* (Fig. 4D, F; 2-way ANOVA interaction *P*>0.05 between *un* and *nkl*, *pkl*, and *ckl*). This indicated that *upsilon* by itself is not involved in mannitol insensitivity. In fact, adding *upsilon* to the *pc* background (=*upc*) did not affect LEH compared with the double mutant *pc* or its WT. In contrast, addition of *kappa*, *lambda*, and *nu* to *pc (=kpc*, *lpc*, *npc)* did enhance LEH on mannitol significantly as compared with WT (18, 23, and 25% respectively; Fig. 4D, F). For the triple mutants in the *un* background adding the remaining four single mutants showed larger LEH compared with the WT (Fig. 4D, F). However, they did not significantly differ from *un* itself (2-way ANOVA interaction *P*>0.05). The single mutants, *kappa* and *nu*, showed a significantly enhanced LEH compared with the WT; 13% and 12%, respectively. Taken together, unequal redundancy is seen between *upsilon* and *nu* with respect to mannitol insensitivity, whereby NU itself is involved in mannitol sensitivity.

### *In-vivo* 14-3-3 pull-down assay in extracts of roots treated with(out) mannitol

Our next step in understanding the LEH phenotypes found was to see whether there is a change in the 14-3-3 interactome when roots are exposed to osmotic stress. Therefore, we performed a 14-3-3 pull-down using soluble proteins extracted from roots that were exposed to 200mM mannitol stress at three different time points: 0min (=control), 10min, and 3h. The mannitol treatment was chosen because it induces osmotic stress and elevation of ACC (Felix *et al.* 2000; Zhao *et al.* 2004; Christmann *et al.* 2005; Christmann *et al.* 2007). In addition, 14-3-3 binding to target proteins is phosphorylation-dependent, and it is known that osmo-stress activated kinase families like SnRK, MAPK, and CDPK are activated within a 10 minute time scale (Ichimura *et al.* 2000; Boudsocq *et al.* 2004; Franz *et al.* 2011). For the pull-down assay nickel-beads were coated with N-terminally His-labelled 14-3-3 KAPPA, LAMBDA, NU, UPSILON, and PHI in equimolar ratios (1:1:1:1:1). The non-phosphorylated peptide R18 was used for specific elution of target proteins by competing with targets that bind to the 14-3-3 binding groove (Supplementary Table S2). Although the R18 elution is specific for 14-3-3 targets it is a competition elution which means that stronger bound proteins may not be eluted. Therefore, a second elution with imidazole was performed to get insight into the stronger binding proteins as well (Supplementary Table S2). Eluted proteins were separated by SDS-PAGE electrophoresis and the identity of the proteins was determined by MS/MS spectrometry. The experiment was conducted three times with independent biological replicas meaning that in the end each treatment has six profiles (3 times R18 and 3 times imidazole). Supplementary Table S3 shows the list of proteins identified in at least two independent samples of the triple pull-down. A total of 73 proteins was identified at different time points. The identified proteins are grouped in three categories according to the following criteria: (i) proteins identified at all-time points (1–21); (ii) proteins disappearing at the 10min and/or 3h time points (22–36); (iii) proteins absent before the stress-treatment (t=0min) and appearing at 10min and/or 3h time point (37–73).

The list includes known 14-3-3 targets or proteins found in other 14-3-3 pull-down assays; e.g. in category 1, nitrate reductase (Bachmann *et al.* 1996; Chang *et al.* 2009; Shin *et al.* 2011) and cytosolic invertase (Chang *et al.* 2009); CRA1 and SESA2 in category 2 (Chang *et al.* 2009); and in category 3 CPK3 (Lachaud *et al.* 2013). There were nine proteins exclusively found in the control plants, indicating that the interaction of these proteins with 14-3-3s is negatively affected by mannitol. A total of 22 proteins were found solely in the 10-min treatment; this indicates that during 10min mannitol treatment, phosphorylation of these 14-3-3 targets occurs. Among these proteins are stress-related proteins like a peroxidase, EARLY-RESPONSIVE TO DEHYDRATION 2, and CATALASE2. Next to the aforementioned 22 proteins found solely after 10min treatment, 15 proteins were only found in samples that were exposed to mannitol for 10min and/or 3h; among these are CPK3, a WD40 repeat family protein and tubulin beta-2/beta-3 chain and tubulin alpha-2/alpha-4 chain. Taken together, the 14-3-3 interactome is markedly affected by mannitol treatment of the roots, which shows that 14-3-3 proteins are involved in osmotic stress adaptation. The mannitol phenotypes of the 14-3-3 mutant plants, as shown in Fig. 4, are therefore likely because the 14-3-3 proteins interact in wild-type plants with some of these targets, changing their overall state. This is lacking in the mutant plants.

## Discussion

In this study we describe the genetic interaction of six non-epsilon 14-3-3 genes in *Arabidopsis*. The phenotypic analysis showed that 14-3-3s are involved in primary root growth under control conditions and under stress conditions. In general, our analysis indicated overlapping functions of these 14-3-3s in root growth. However, we also detected phenotypic differences that are consistent with isoform-specific functions and potential antagonistic functions between the genes tested. In addition, the 14-3-3 root interactome markedly responded to mannitol application.

### 14-3-3s are positive regulators of primary root growth under control conditions

14-3-3 proteins have been linked to root growth, notably to primary root (PR) length (Mayfield *et al.* 2012; Li *et al.* 2013) The root assay revealed shorter PR length compared with WT for the three quadruple mutants and for six triple mutants (*pkl*, *kun*, *pun*, *cun*, *npc*, and *upc*) and a time-dependent delay in growth. The primary root is formed during embryogenesis and examples are known for *Arabidopsis* mutants that show shorter primary roots related to meristem cell differentiation and size (Aida *et al.* 2004; Lucas *et al.* 2011). However, preliminary data suggest that our 14-3-3 mutants are not affected in root meristem integrity (data not shown). The shorter PR and the change in growth rates seen at different time points in the 14-3-3 mutants could be explained by a change in sugar levels, hormonal synthesis/signalling, or nutrient levels (Shin *et al.* 2007; Shin *et al.* 2011). 14-3-3 proteins have been implicated in all the aforementioned processes. It could very well be that removing 14-3-3s results in the plant becoming imbalanced in the above-mentioned pathways. For example, in 14-3-3 loss-of-function mutants, some 14-3-3 targets will become less active when 14-3-3 interaction increases their activity, e.g. CPK1 (Camoni *et al.* 1998), or become more active when 14-3-3 inhibits their activity, e.g. NR (MacKintosh *et al.* 2001). These differences in 14-3-3 target activity status may cause the change in growth rate seen in the root assay. A time-dependent effect has been seen in other genetic interaction studies as well. The study using *Arabidopsis thaliana* histidine phosphotransfer proteins (AHPs), which are involved in cytokinin signalling, showed that primary root elongation between WT and *ahp1*,*2*,*3*,*4*,*5* was normal in the first days and thereafter growth was slowed down (Hutchison *et al.* 2006). Another example is the phenotype of the 14-3-3 *mu-1* mutant, where the roots of the mutant plants showed no further increase in root greening after 10 days similarly to the root of WT plants (Mayfield *et al.* 2012). What the exact mechanism of reduced root elongation is remains unknown and further research is needed.

### 14-3-3s are negative regulators in abiotic stress

The 14-3-3 mutants show different responses to the mannitol and ACC treatment, which could indicate that: (i) there is isoform specificity or different heterodimers are involved in different stress perceptions/signalling, or (ii) the pathways that regulate LEH are differently regulated by different stresses. 14-3-3s are found to stabilize members of the early responsive transcription factor family AREB/ABF. The C-terminal tip (T451) of AtABF3 is phosphorylated by SnRK2.6/OST1, a kinase that is activated by ABA and mannitol (Boudsocq *et al.* 2004; Sirichandra *et al.* 2010).

In mannitol there seems to be antagonistic effects between 14-3-3 isoforms. The combination of the single mutants *upsilon* and *nu* (the double mutant *un*) shows a larger LEH than WT on mannitol. Adding *upsilon* to the *kl*- or the *pc*-background shows an LEH comparable to WT whereas adding *nu* to these backgrounds shows a larger LEH compared with WT. These data indicate antagonistic effects among the 14-3-3 isoforms. A similar effect was seen in a genetic interaction of ARR genes, which shows that highly similar genes can have different effects and suppress each other’s gene function (To *et al.* 2004). The single mutant *arr5* showed a reduced petiole size. The mutant of its closely related gene *ARR6* was indistinguishable from WT, whereas both the double mutant *arr5*,*6* and quadruple mutant arr*5*,*6*,*8*,*9* are indistinguishable from WT. This indicates that *arr6* suppresses the *arr5* reduced rosette size mutant phenotype (To *et al.* 2004).

The ACC phenotype of *kl* is intriguing because *kl* is the only isoform combination that shows reduced ACC sensitivity. For mannitol, the quadruple mutants all show larger LEH compared with WT whereas for ACC only the higher order mutants in the *kl* background or higher order mutants containing *kappa* or *lambda* show larger LEH compared with WT plants. Cell elongation is driven by cell wall acidification through the activity of the plasma membrane H^+^-ATPase which activates cell wall expansins. A further reduction in the cell expansion occurs through peroxidase-mediated cross-linking activity in the cell wall. Peroxidases have been identified in 14-3-3 pull-down studies (Supplementary Table S3, available at JXB online, and Shin *et al.* 2011). For 14-3-3s to be involved in peroxidase-mediated cross-linking they need to reside outside the cell, which has been demonstrated (Voigt and Frank, 2003; Wen *et al.* 2007). It is tempting to speculate that the 14-3-3 *kl* mutant shows reduced ACC sensitivity because peroxidases cannot interact with these 14-3-3 isoforms. In addition, the peroxidase (PER69), found in our pull-down study, has been identified as a cell wall protein in *Arabidopsis* (Irshad *et al.* 2008). We conclude that 14-3-3 shows isoform-specific redundancy in the ACC phenotyping, because only mutants containing *kappa* or *lamda* or *kl* show a larger LEH compared with WT.

Although there is evidence of plant 14-3-3 transcripts being up-regulated during abiotic stress, no indication was found here for *Arabidopsis* 14-3-3s (Fig. S2, available at JXB online) (Aksamit *et al.* 2005; Xu and Shi, 2006). Therefore, a pull-down assay using recombinant 14-3-3s and total protein extract from roots treated with 200mM mannitol was performed. A total of 37 proteins (out of 73) were identified exclusively during mannitol treatment. Included in the list are known targets or proteins previously found in 14-3-3 pull-down assays, such as nitrate reductase (Bachmann *et al.* 1996; Chang *et al.* 2009; Shin *et al.* 2011), and CPK3 (Lachaud *et al.* 2013). Members of the universal stress protein, such as PHOS32 and PHOS34 are known to be involved in abiotic stress and are known targets of MAPK MPK3, and MPK6 (Merkouropoulos *et al.* 2008). In addition, a MAPKKK, VH1-INTERACTING KINASE (VIK), was found in the pull-down assay. It is noteworthy that the protein list from a 14-3-3 pull-down is a collection of primary 14-3-3 targets as well as secondary proteins (which are in complex with the primary target); therefore not all proteins in the list are primary 14-3-3 targets and more research is needed to establish which proteins are primary targets and which ones are secondary targets. In addition, although 14-3-3 targets were specifically eluted with the R18 peptide and 14-3-3 was released from the beads by imidazole, we cannot exclude the presence of non-specifically bound proteins. The pull-down assay using five 14-3-3 isoforms showed 22 proteins found solely at 10 minute mannitol treatment. As 14-3-3 binds phosphorylated targets this result indicates that phosphorylation of 14-3-3 targets is rapidly increased through the activation of osmo-stress activated kinases (Ichimura *et al.* 2000; Boudsocq *et al.* 2004).

## Outlook

This study shows isoform specificity and redundancy among six 14-3-3 isoforms in root phenotypes. Higher order loss-of-function mutants were generated according to the position within the phylogenetic tree. However, the phylogenetic tree does not reflect the subcellular localization and tissue specificity of the 14-3-3 isoforms. For example, differences in tissue 14-3-3 localization can be seen between two closely related 14-3-3 isoforms KAPPA and LAMBDA. Both isoforms are nuclear localized in trichomes, but in stomata KAPPA is nuclear localized whereas LAMBDA is restricted to the edges of the stomata (Paul *et al.* 2005). Further research will focus on the subcellular localization of these six 14-3-3 genes. In addition, extra double mutant lines will be made to investigate the possibility that only two genes are involved in a phenotype, e.g. *kp* and *lp* in the shorter root phenotype of *klp*, or *kn* in the mannitol LEH phenotype. In conclusion, the mutants generated in this study indicate that 14-3-3s are involved in regular primary root growth and growth under osmo-stress, but further research is needed to conclude whether subcellular localization is at the basis of these phenotypes.

## Supplementary data

Supplementary data are available at *JXB* online

Table S1. Primers and PCR conditions.

Table S2. Mass spectrometry data.

Table S3. Mass spectrometry data overview of 73 proteins found.

Figure S1. Transcript PCRs of the higher order mutants.

Figure S2. *In silico* microarray data of 14-3-3 expression under different stresses.

Supplementary Data
