# Supplementary material for: Higher order Arabidopsis 14-3-3 mutants show 14-3-3 involvement in primary root growth both under control and abiotic stress conditions
Source: J Exp Bot. 2014 Sep 4;65(20):5877–88. doi: 10.1093/jxb/eru338 (PMC4203132; doi:10.1093/jxb/eru338)
Supplement: Supplementary Data [file supp_65_20_5877__index.html]

Higher order Arabidopsis 14-3-3 mutants show 14-3-3 involvement in primary root growth both under control and abiotic stress conditions — Higher order Arabidopsis 14-3-3 mutants show 14-3-3 involvement in primary root growth both under control and abiotic stress conditions — Supplementary Data 

# Higher order *Arabidopsis* 14-3-3 mutants show 14-3-3 involvement in primary root growth both under control and abiotic stress conditions

## Supplementary Data

Data files

**Files in this Data Supplement:**

- Supplementary Data - Supplementary Data
- Supplementary Data - Supplementary Data
- Supplementary Data - Supplementary Data
- Supplementary Data - Supplementary Data
