# Supplementary material for: Higher order Arabidopsis 14-3-3 mutants show 14-3-3 involvement in primary root growth both under control and abiotic stress conditions
Source: J Exp Bot. 2014 Sep 4;65(20):5877–88. doi: 10.1093/jxb/eru338 (PMC4203132; doi:10.1093/jxb/eru338)
Supplement: Supplementary Data [file supp_eru338_jexbot125666_file001.pdf]

Higher order *Arabidopsis* 14-3-3 mutants show 14-3-3 involvement in primary root growth both under control and abiotic stress conditions.

P.J.M. van Kleeff, N. Jaspert, K.W. Li, S. Rauch, C. Oecking and A.H. de Boer

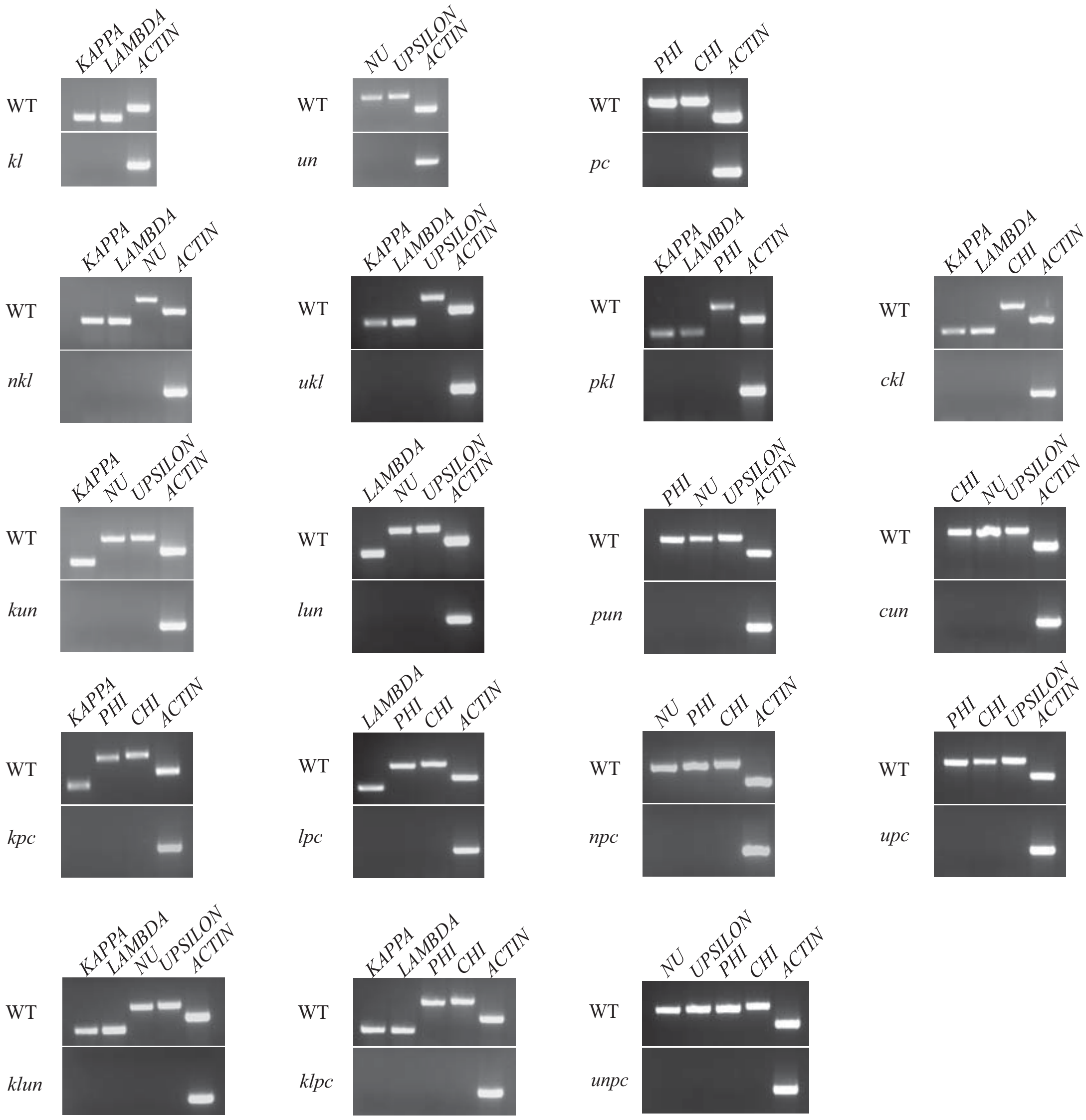

Figure S1. Transcript PCRs of the higher order 14-3-3 mutants.

Total RNA was isolated and transcripts were detected using RT-PCR. The mutated 14-3-3 genes show no full length transcripts and are therefore considered being null mutants.

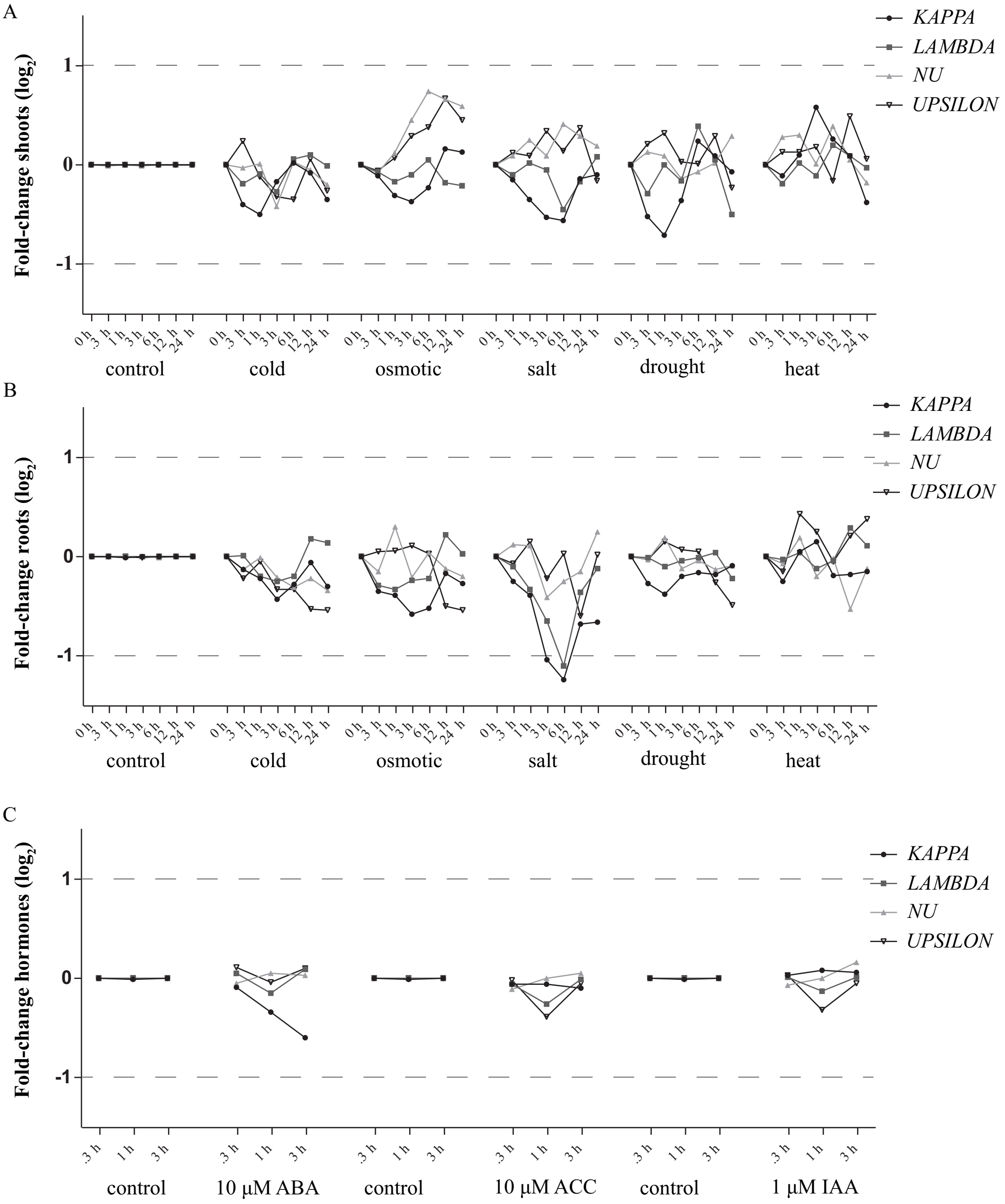

Figure S2. Expression patterns of 14-3-3 in *Arabidopsis* (Col-0) under various abiotic and exogenous applied hormones from publicaly avialable microarray data.

(A) and (B) treatments used; Cold treatment at 4°C, Osmotic is 300 mM mannitol,Salt is 150 mM NaCl, Drought 14 min in an air stream with loss of approximately 10% fresh weigght, Heat 3 h 38°C and recovered at 25°C. (A) At14-3-3 expression in shoots of 18 days after sowing during abiotic stress. (B) At14-3-3 expression in roots of 18 days after sowing during abiotic stress. (C) At14-3-3 expression in 7 day old seedlings stressed with 10 µM ACC, 1 µM IAA and 10 µM ABA. Dashed lines indicate a 2-fold change in 14-3-3 expression
